# Heterologous expression of a rice *miR395* gene in *Nicotiana tabacum* impairs sulfate homeostasis

**DOI:** 10.1038/srep28791

**Published:** 2016-06-28

**Authors:** Ning Yuan, Shuangrong Yuan, Zhigang Li, Dayong Li, Qian Hu, Hong Luo

**Affiliations:** 1 Department of Genetics and Biochemistry, Clemson University, 105 Collings street, 110 Biosystems Research Complex, Clemson, South Carolina, 29634-0318, USA

## Abstract

Sulfur participates in many important mechanisms and pathways of plant development. The most common source of sulfur in soil –SO_4_^2−^– is absorbed into root tissue and distributed into aerial part through vasculature system, where it is reduced into sulfite and finally sulfide within the subcellular organs such as chloroplasts and mitochondria and used for cysteine and methionine biosynthesis. MicroRNAs are involved in many regulation pathways by repressing the expression of their target genes. *MiR395* family in *Arabidopsis thaliana* has been reported to be an important regulator involved in sulfate transport and assimilation, and a high-affinity sulphate transporter and three ATP sulfurylases (ATPS) were the target genes of *AthmiR395* (*Arabidopsis thaliana miR395*). We have cloned a *miR395* gene from rice (*Oryza sativa*) and studied its function in plant nutritional response. Our results indicated that in rice, transcript level of *OsamiR395* (*Oryza sativa miR395*) increased under sulfate deficiency conditions, and the two predicted target genes of *miR395* were down-regulated under the same conditions. Overexpression of *OsamiR395h* in tobacco impaired its sulfate homeostasis, and sulfate distribution was also slightly impacted among leaves of different ages. One sulfate transporter (SULTR) gene *NtaSULTR2* was identified to be the target of *miR395* in *Nicotiana tobacum*, which belongs to low affinity sulfate transporter group. Both *miR395* and *NtaSULTR2* respond to sulfate starvation in tobacco.

As a rudimental and essential element, sulfur is one of the six macronutrients required for plant growth and participates in many important physiological and biochemical processes. In nature, sulfur exists in both inorganic and organic forms, and sulfate (SO_4_^2−^) is the most common inorganic source of sulfur plants acquire from soil.

The sulfate absorption and assimilation pathway in plants is a complex system. In the very beginning, sulfate is absorbed into root tissue. Except for a small amount of sulfate stored in vacuole of root cells, the majority of them are distributed into aerial part through vasculature system. Upon transfer into subcellular organs such as chloroplasts and mitochondria in cells of aerial part, the sulfate is reduced into sulfite, then sulfide used for the synthesis of cysteine and methionine, two amino acids that play a pivotal role in sulfate assimilation pathway^1^, and essential for supporting many important redox reactions in plants. The reduced form of the cysteine could function as an electron donor and its oxidized form could act as an electron acceptor.

Given the important role sulfur plays in plant growth and development, its deficiency (−S) would cause severe problems to plants, resulting in decreased plant yields and quality^2^. To genetically improve plant sulfate uptake and utilization under −S conditions, it is essential to fully understand the functions of the genes encoding sulfate transporters and other important components involved in sulfate assimilation pathways^2^.

Over the course of the past 20 years, essential genes involved in sulfate uptake, distribution and assimilation pathways have been identified and well-studied in different plant species. *Shst 1*, *Shst 2* and *Shst 3* were the first sulfate transporter genes cloned from *Stylosanthes hamate* responsible for initial sulfate uptake and internal transport^3^. In *Arabidopsis*, since the cloning of the first sulfate transporters, AST56 and AST68 two decades ago^4^, at least 12 *Arabidopsis* sulfate transporters belonging to five different groups have been identified^5^. These include two high-affinity sulfate transporters SULTR1;1 and SULTR1;2 responsible for uptake of sulfate from soil^6^,^7^ low-affinity sulfate transporters SULTR2;1 and SULTR2;2 responsible for internal transport of sulfate from root to shoot^7^, SULTR3;5, the function partner of the SULTR2;1 that facilitates the influx of sulfate^8^, and SULTR4;1 and SULTR4;2 involved in distribution of sulfate between *Arabidopsis* vacuoles and symplastic^9^. The *ORYsa;Sultr1;1* and *ORYsa;Sultr4;1* are the first two sulfate transporters cloned from rice in early 2000 s^10^, followed by the identification of additional 12 sulfate transporters^11^.

ATP sulfurylase (ATPS) catalyzes the synthesis of the essential metabolic intermediate, adenosine 5′-phosphosulfate (APS), and this step is the branch point of the sulfate assimilation pathway followed by the synthesis subpathways of either cysteine or other sulfated compounds. ATPS has been extensively studied for the past decade because of its important role in the sulfate assimilation pathway^12^,^13^,^14^,^15^. *SULTR* or *ATPS* gene families would be the ideal targets for genetic modification to increase the efficiency of plant sulfate uptake and assimilation under −S conditions. It is therefore important to understand how they are regulated in plants.

MicroRNAs (miRNAs) are short non-coding RNAs with only 20–24 nt, regulating many metabolisms in the post-transcriptional level by repressing translation of their target genes. In plants, with the help of RISC (RNA inducing silence complex), mature miRNA could form near-perfect pairs with its complementary sequences of the mRNA target, followed by cleavage of the base-pairing region and degradation of the transcripts^16^. Among thousands of identified *miRNAs*, *miR395* family in *Arabidopsis* has previously been reported to be an important regulator involved in sulfate transport and assimilation^17^,^18^,^19^. The targets of *AthmiR395* (*Arabidopsis thaliana miR395*) are sulfate transporter genes and *ATPS*, such as high-affinity sulfate transporter gene, *AthSULTR2:1* and ATP sulfurylase genes, *AthATPS*1, 3, and 4^19^,^20^,^21^,^22^.

The divergence of monocot and dicot plants occurred at 200 million years ago^23^, but the miRNA-mediated gene regulation mechanism has an even longer history, which is more than 425 million years^24^. These facts suggest that monocot and dicot plants should have a similar miRNA-mediated gene regulation mechanism and conserved miRNA families sharing the same gene ancestors and regulating the same biological events. Research for the past two decades has led to the identification of 21 miRNA families including many well-studied ones such as miR156 and miR399 that seem to be highly conserved between monocots and dicots^25^. *MiR395* is also on the list, but experimental support is still lacking.

Sequences of mature *miR395* are highly conserved between model plant, *Arabidopsis* and crop species. Understanding the role *miR395* plays in important food crops would allow development of novel biotechnology approaches to genetically engineer these plants for ameliorated nutrient uptake and utilization, improving plant growth, yield and agricultural productivity. We have cloned pri-*OsamiR395h* (*Oryza sativa miR395*) from rice (*Oryza sativa*) and studied its function in plant nutritional response. Our results showed that transcript level of *OsamiR395* increased under −S condition accompanied with down regulation of its two predicted target genes. Overexpression of pri-*OsamiR395h* in tobacco (*Nicotiana tobacum*) impaired its sulfate homeostasis. Sulfate distribution was also slightly impacted between leaves of different ages in transgenic plants. One potential target gene of *miR395* named *NtaSULTR2* was identified in tobacco (*Nicotiana tobacum*), which encodes a sulfate transporter. The expression of both endogenous *NtamiR395* (*Nicotiana tobacum miR395*) and *NtaSULTR2* was significantly induced under low sulfate conditions in tobacco leaf tissues, but the expression level of *NtaSULTR2* was inversely correlated to that of *NtamiR395* under different sulfate conditions in root tissues. These results indicate that *OsamiR395* responds to −S by inducing degradation of two target genes, and pri-*OsamiR395h* can function in dicot plant tobacco and impact its sulfate transportation and distribution. As the first target gene of *miR395* identified in tobacco, *NtaSULTR2* encodes a sulfate transporter belonging to the low-affinity group.

## Results

### Sulfate regulates the expression of *OsamiR395* and its target genes

**Figure 1:**
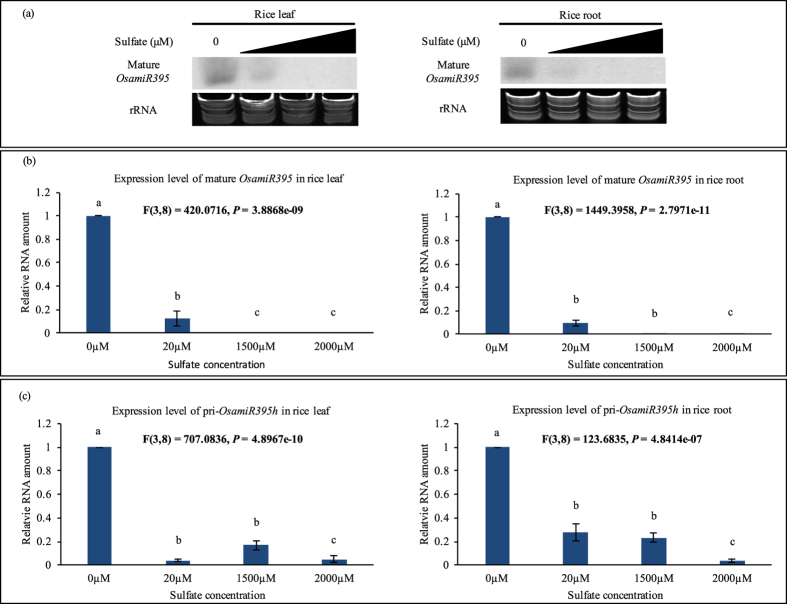
Sulfate deficiency induces accumulation of *OsamiR395* in rice. (**a**) Small RNA northern blotting analysis of mature *OsamiR395* under different sulfate concentrations. Total RNA samples were prepared from leaf and root tissues of two weeks old rice grown in N6 medium with 0, 20, 1500 or 2000 μM (NH^4+^)_2_SO_4_ and used for small RNA northern blotting analysis. Antisense oligonucleotides of *OsamiR395* was labeled with γ-[^32^P]ATP and used as probe to detect the transcript level of mature *OsamiR395*. rRNA was used as a loading control. (**b**) Stem-loop real-time PCR analysis of mature *OsamiR395* under different sulfate concentrations. Total RNA samples were prepared as in (**a**) and used for stem-loop real-time PCR analysis. *OsaSIZ1* was used as a reference gene. Data are presented as means of three technique replicates, error bars represent SD (n = 3). (**c**) Real-time PCR analysis of rice pri-*OsamiR395h* under different sulfate concentrations. Total RNA samples were prepared as in (**a**) and used for real-time PCR analysis. *OsaSIZ1* was used as a reference gene. Data are presented as means of three technique replicates, error bars represent SD (n = 3). The statistically significant difference between groups was determined by one-way ANOVA (F(df_between_, df_within_) = F ration, *p* = p-value, where df = degrees of freedom). Means not sharing the same letter are statistically significantly different (P < 0.05).

According to previous research and miRNA database (http://mirbase.org), 24 family members belonging to four clusters comprise *OsamiR395* family^26^. The sequence of mature *OsamiR395* is highly conserved while the pre-microRNA sequences are divergent. It has previously been demonstrated in *Arabidopsis* that mature *AthmiR395* transcript accumulates under sulfur-limited conditions^18^,^19^,^27^. To investigate whether *OsamiR395* also responds to low sulfate conditions as its counterpart in *Arabidopsis*, transcript level of *OsamiR395* in two weeks old rice plants grown in N6 solid medium supplemented with different concentrations of sulfate was analyzed. Both northern blotting and stem-loop RT-PCR results showed that the transcripts of mature *OsamiR395* accumulated under low sulfate conditions (0 and 20 μM SO_4_^2−^), but declined significantly under sulfate-adequate conditions (1500 and 2000 μM SO_4_^2^, Fig. 1a,b).

In plant nucleus, *miRNA* gene is first transcribed into a long pri-*miRNA*, which is then processed into pre-*miRNA* and finally mature *miRNA* that is later translocated by HASTY into cytoplasm and induces the degradation of its target gene(s). To further understand whether *OsamiR395* is regulated at the transcription level or post-transcription level, real-time PCR experiment was conducted to investigate the transcript level of *pri-OsamiR395h* in two weeks old rice plants grown in N6 solid medium supplemented with 0, 20, 1500 or 2000 μM SO_4_^2−^. Real-time PCR results showed that excess sulfate could repress the accumulation of pri-*OsamiR395h* transcript (Fig. 1c). Conversely, the transcription level of pri-*OsamiR395h* increased significantly under sulfate deficient conditions (0 and 20 μM SO_4_^2−^, Fig. 1c). Transcript levels of pri- and mature *OsamiR395* exhibit the same trend under sulfate starvation stress, indicating that *OsamiR395* expression is transcriptionally regulated by sulfate. Sulfate starvation stress induces the expression of pri-*OsamiR395h*, leading to the production of more mature *OsamiR395* transcripts.

**Figure 2:**
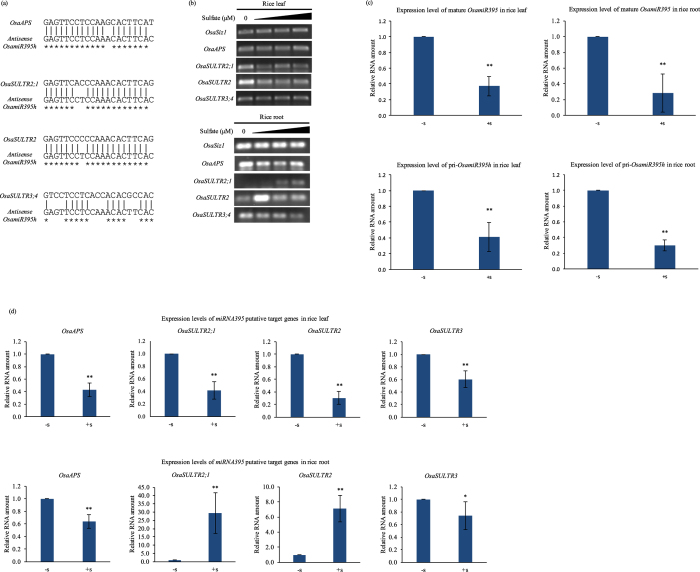
Predicted target *OsaSULTR1* and *OsaSULTR2* exhibit opposite expression patterns to that of the *OsamiR395* in rice root. (**a**) Target sites of the four putative *OsamiR395* target genes in rice. The target sites were compared with the complementary sequence of mature *OsamiR395h*. Asterisks indicate the identical sequences. (**b**) RT-PCR analysis of expression levels of the *OsamiR395* putative targets. Total RNA samples used for RT-PCR were extracted from leaf and root tissues of two weeks old rice grown in N6 medium with 0, 20, 1500 or 2000 μM (NH^4+^)_2_SO_4_ and used for RT-PCR analysis. *OsaSIZ1* was used as a reference gene. Experiment was repeated three times. (**c**) Stem-loop real-time RT-PCR analysis of mature *OsamiR395* and real-time RT-PCR analysis of *pri-OsamiR395h*. Total RNA samples were prepared from leaf and root tissues of two weeks old rice grown in regular N6 medium (+S) or N6 medium without SO_4_^+^ (−S) and used for RT-PCR analysis. *OsaSIZ1* was used as a reference gene. (**d**) Real-time RT-PCR analysis was also conducted to determine the expression levels of the *OsamiR395* putative targets in rice leaves and roots. Total RNA samples were prepared as in (**c**) and used for real-time RT-PCR analysis. *OsaSIZ1* was used as a reference gene. For (**c,d**), data are presented as means of two independent biological replicates and three technical replicates, error bars represent SD (n = 6). Asterisks indicate the significant differences between expression levels under −S and +S conditions. P < 0.05 is marked as*, P < 0.01 is marked as**.

Computational analysis of the rice genome sequences leads to the identification of four putative targets of *OsamiR395*, including one *ATPS* and three sulfate transporter genes, *OsaSULTR2;1*, *OsaSULTR2* and *OsaSULTR3;4* (Fig. 2a)^17^,^27^. RT-PCR results indicated that *OsaATPS* did not exhibit any responses in both roots and leaves under −S stress. *OsaSULTR3;4* did not respond to sulfate treatment in leaves either, but was down-regulated in roots with the increasing sulfate concentrations, exhibiting similar expression pattern as *OsamiR395* (Fig. 2b). *OsaSULTR2;1* and *OsaSULTR2* genes were both down-regulated in leaves with the increasing sulfate concentrations (Fig. 2b), similar to the expression pattern of *OsmiR395* in response to sulfate treatment (Fig. 1). On the contrary, they were both up-regulated in roots in response to increasing sulfate concentrations (Fig. 2b). It should be noted that *OsaSULTR2* exhibited the highest induction under 20 μM sulfate, suggesting that other regulation machineries may also participate in the regulation of the *OsaSULTR2* gene under this particular condition. These results support the hypothesis that *OsaSULTR2;1* and *OsaSULTR2* are the putative target genes of, and regulated by *OsamiR395* in rice roots. In rice leaves, however, *OsamiR395*-mediated transcript cleavage of the *OsaSULTR2;1* and *OsaSULTR2* genes may not be able to take place due to their non-overlapping tissue-specific expression. Instead, there may exist some other mechanisms regulating the expression of *OsaSULTR2;1* and *OsaSULTR2*. This is also likely the case for *OsaSULTR3;4* in roots. Similar phenomena have previously been observed in *Arabidopsis*^18^,^19^. It should be noted that there are multiple mismatches in the *OsamiR395* target sequence of the *OsaSULTR3;4* (Fig. 2a). This raises the question of whether or not *OsaSULTR3;4* is indeed the true target of *OsamiR395*.

To confirm the results of semi-quantitative RT-PCR, real-time RT-PCR was conducted to determine the expression levels of *OsamiR395* and its putative targets in rice under −S condition (N6 medium without sulfate) and +S condition (regular N6 medium). Real-time PCR results were consistent with that of the semi-quantitative RT-PCR. In both leaves and roots, pri- and mature *OsamiR395* were up-regulated under −S condition (Fig. 2c). Among the four putative target genes, only *OsaSULTR2;1* and *OsaSULTR2* were significantly down-regulated in rice roots under −S condition, exhibiting opposite trend of expression to *OsamiR395* (Fig. 2d), in agreement with the results obtained by semi-quantitative RT-PCR and supporting the notion that *OsaSULTR2;1* and *OsaSULTR2* are the putative targets of *OsamiR395* in rice roots.

### Expression of the *OsamiR395* and its target genes is spatiotemporally regulated

**Figure 3:**
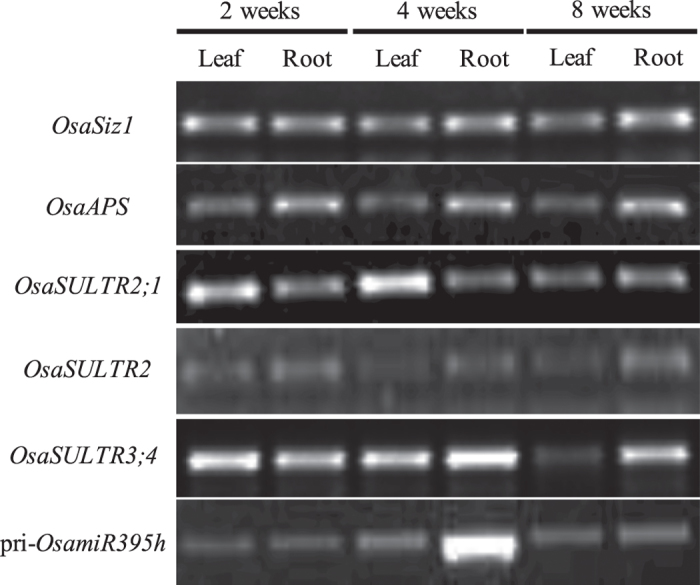
Expression level of pri-*OsamiR395h* and its target genes in rice leaf and root tissues at different developmental stages. Total RNA samples were prepared from leaf and root tissues of rice harvested at indicated time points and used for RT-PCR analysis. *OsaSIZ1* was used as a reference gene. Experiment was repeated three times.

Besides the response of *OsamiR395* and its targets to sulfate starvation stress, we also investigated the expression patterns of *OsamiR395* and its target genes in different developmental stages and tissues. To this end, we particularly focused on the primary miRNA level for one of the rice *OsamiR395* genes, *OsamiR395h* and the expression of its putative target genes in both roots and leaves at different developmental stages under normal growth conditions. The RT-PCR results showed that the expression of pri-*OsamiR395h* was strongly induced only in the roots of the four weeks old plants, but otherwise remained very low in both roots and leaves in any other developmental stages (Fig. 3).

The expression of the *ATPS* again was quite stable in both tissues throughout the rice development, but an elevated expression level in roots was observed compared to that in leaves (Fig. 3). The expression levels of the three sulfate transporter genes were variable, but none of them was inversely correlated with that of the *OsamiR395h* (Fig. 3).

### Heterologous expression of pri-*OsamiR395h* in *Nicotiana tabacum*

**Figure 4:**
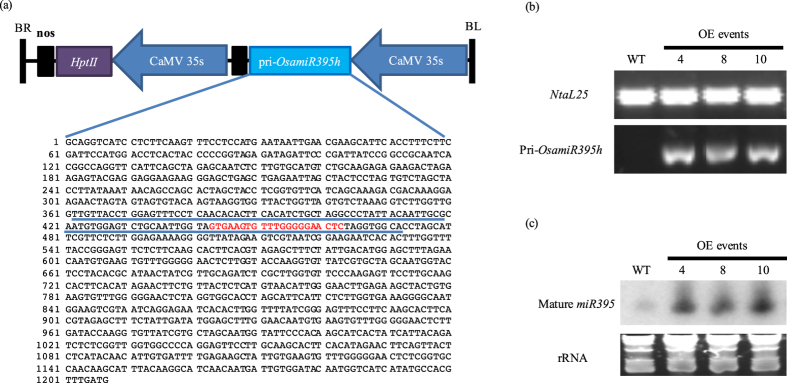
Heterologous expression of pri-*OsamiR395h* in *Nicotiana tabacum*. (**a**) The Schematic diagram of rice pri-*OsamiR395h* overexpression construct. Rice pri-*OsamiR395h* sequence containing stem-loop structure of *OsamiR395h* was cloned from rice genomic DNA and put under the control of the CaMV35S promoter. The *hptII* gene driven by CaMV35S promoter was used as selectable maker. The pre-*OsamiR395h* sequence was underlined. Sequence emphasized with red color indicates the mature *miR395h*. LB, Left border; RB, right border. (**b**) RT-PCR analysis of pri-*OsamiR395h* expression in wild type and three transgenic tobacco lines. Total RNA samples were prepared from two weeks old wild type and transgenic tobacco plants grown in MS medium. *NtaL25* was used as reference gene. (**c**) Small RNA northern blotting analysis of mature *miR395* transcripts in wild type and three transgenic tobacco lines. Total RNA samples were prepared from two weeks old wild type and transgenic tobacco plants grown in MS medium. rRNA was used as loading control. WT: wild type plant. OE: overexpression line.

To further study the role *OsamiR395* plays in sulfate transportation and distribution, we generated a chimeric DNA construct containing the pri-*OsamiR395h* sequence driven by the CaMV35S promoter (Fig. 4a). This construct was then introduced into tobacco (*Nicotiana tabacum*) to produce a total of 10 independent transgenic events. RT-PCR analysis suggested rice pri-*OsamiR395h* was successfully expressed in tobacco (Fig. 4b), and small RNA northern blotting result suggested rice pri-*OsamiR395h* was successfully processed into mature *miRNA* (Fig. 4c). The detection of tobacco endogenous mature *NtamiR395* in northern blotting indicated that mature *NtamiR395* shares a highly conserved sequence with its rice homolog. Three independent transgenic events were selected for further analysis.

### Overexpression of the rice *pri-OsamiR395h* impairs sulfate homeostasis and leads to retarded plant growth in transgenic tobacco

**Figure 5:**
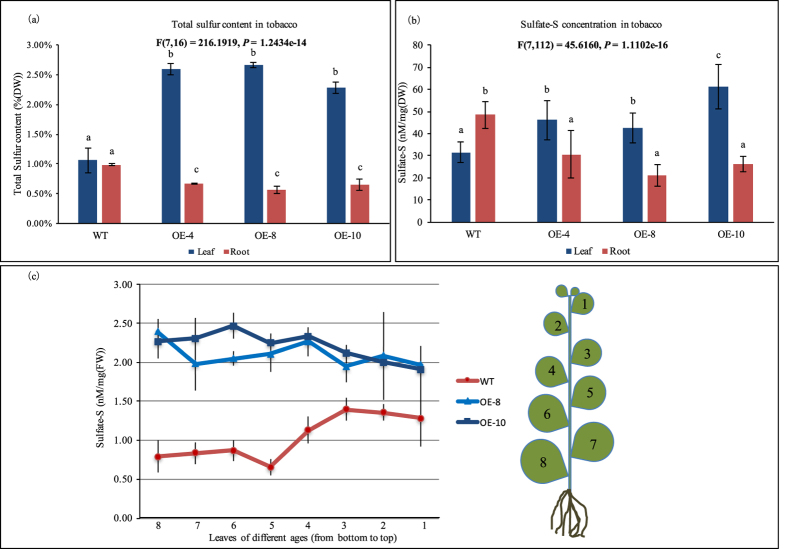
Overexpression of pri-*OsamiR395h* impacts tobacco sulfate transportation and distribution. (**a**) Statistical analysis of total sulfur in leaf and root tissues. Samples were harvested from four weeks old wild type and three transgenic tobacco lines. Data are presented as means of three biological replicates contains mixed samples from five biological replications, error bars represent SD (n = 3). (**b**) Statistical analysis of sulfate-S concentrations in leaf and root tissues. Samples were harvested from four weeks old wild type plants and three transgenic tobacco lines. Data are presented as means of fifteen biological replicates, error bars represent SD (n = 15). The statistically significant difference between groups was determined by one-way ANOVA (F(df_between_, df_within_) = F ration, *p* = p-value, where df = degrees of freedom). Means not sharing the same letter are statistically significantly different (P < 0.05). (**c**) Statistical analysis of sulfate concentration in tobacco leaves of different ages. Leaves of 12 weeks old wild type and three transgenic tobacco lines were harvested in the positions as indicated in the figure. Data shown are an average of three biological replicates, error bars represent SD (n = 3). DW: dry weight. FW: fresh weight. WT: wild type.

It has previously been shown that overexpression of *AthmiR395* in *Arabidopsis* impairs its sulfate distribution and assimilation^19^. To evaluate the impact of the *OsamiR395* in tobacco sulfate metabolism and plant development, we first measured the total sulfur contents in transgenic tobacco plants and wild type (WT) controls. Not surprisingly, the total leaf sulfur content of all the transgenic lines was 2.16 to 2.50 times higher than that in WT controls. On the contrary, the root sulfur content in transgenic lines was 32% to 42% less than that in WT controls (Fig. 5a).

Next, we determined the sulfate-S (sulfate-sulfur) concentration in WT and transgenic plants. Again, the difference in sulfate-S concentrations between transgenics and WT controls was similar to that of the total sulfur contents. In transgenic leaf tissues, the sulfate-S concentration was 1.35 to 1.96 times higher than that in WT leaves, whereas in roots, transgenics had 38% to 57% less sulfate than WT controls (Fig. 5b). This result indicated that the high-level of *miR395* accumulation in transgenic plants impacts the uptake and transportation of sulfur and sulfate.

**Figure 6:**
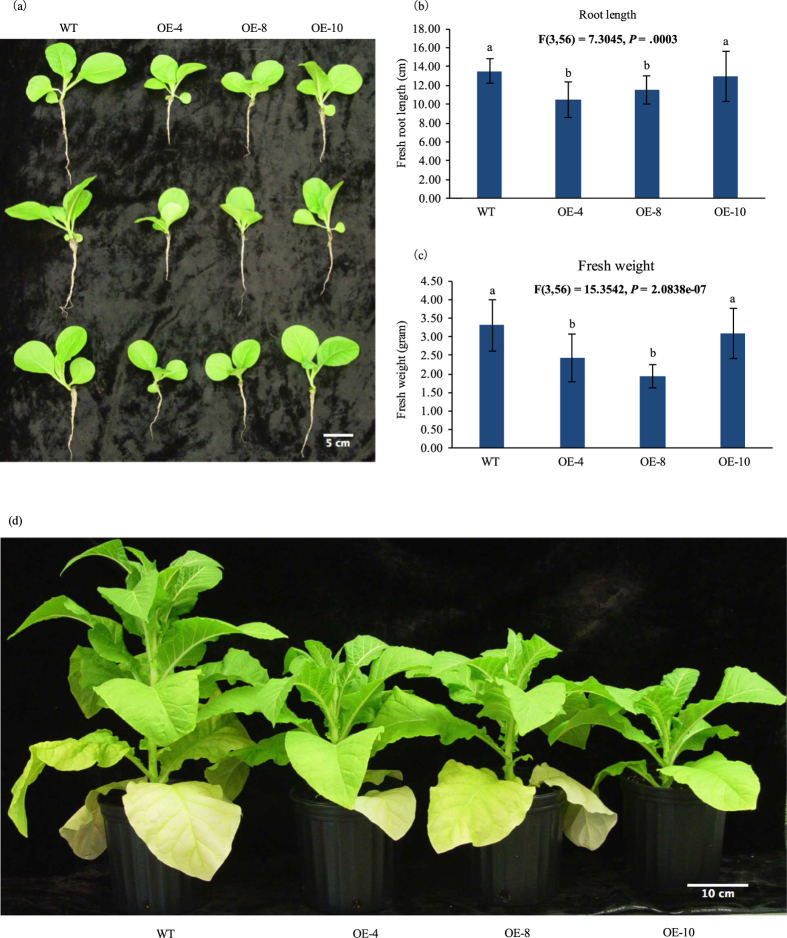
Overexpression of pri-*OsamiR395h* leads to retarded growth of transgenic tobacco. Wild type and transgenic tobacco were grown in soil under 16 h light/8 h dark in greenhouse. Photos were taken (**a**) four weeks and (**d**) seven weeks after seed germination. Representative plants were shown. (**b**) Root length and (**c**) fresh weight of wild type and transgenic tobacco were measured. Data are presented as means of fifteen biological replicates, error bars represent SD (n = 15). The statistically significant difference between groups was determined by one-way ANOVA (F(df_between_, df_within_) = F ration, *p* = p-value, where df = degrees of freedom). Means not sharing the same letter are statistically significantly different (P < 0.05). WT: wild type plant. OE: overexpression line.

Similar to a previous report in *Arabidopsis* that overexpression of *AthmiR395* represses the expression of sulfate transporter gene *AthSULTR2;1* and causes impaired sulfate distributions between leaves of different ages^19^, we also observed that leaf sulfate distribution patterns are different between transgenic tobacco plants and WT controls (Fig. 5c). Because sulfate or sulfur compounds could be transported from old to young leaves under normal or sulfate-adequate conditions^28^, sulfate accumulation in young leaves should be higher than that in old ones as observed in WT control plants (Fig. 5c). Contrary to this, transgenic tobacco plants accumulate fewer sulfates in younger leaves than in older ones (Fig. 5c), indicating that sulfate delivery pathway is impaired in transgenics, which is most likely one of the consequences caused by repressed expression of sulfate transporter genes. Furthermore, compared with WT controls, transgenic tobacco exhibited retarded growth (Fig. 6a,d). As shown in Fig. 6b,c, one-month-old transgenic plants displayed shorter root and less fresh weight than wild type controls, a similar phenotype observed in transgenic *Arabidopsis* overexpressing *AthmiR395*^19^. The slow-growth phenotype of transgenic plants suggests that the expression of *ATPS* may also have been strongly repressed in transgenics, resulting in interrupted sulfate assimilation pathway and consequently retardation in plant growth because of the shortage of cysteine and other sulfate metabolic products.

### Identification of *miR395* target gene in tobacco

To understand how the excess *miR395* impacts tobacco sulfate homeostasis at the molecular level, we sought to identify putative new target genes of *miR395* using two approaches^29^. We first used the DNA sequences of the *Arabidopsis SULTR2;1* and *ATPS* genes to blastn against the *Nicotiana tabacum* EST sequences. All the DNA sequences with high similarity (identity of more than 70%) were used to do alignment with complementary sequence of the mature *OsamiR395h*. The following criteria were used to determine the predicted target sequences with minor modifications: (1) No more than four mismatches between *OsamiR395h* and its predicted target genes; (2) No more than two constitutive mismatches between *OsamiR395h* and its predicted target genes; (3) No mismatches between position 10 and 11; (4) No gaps between *OsamiR395h* and its predicted target genes^29^. Besides, we also designed primers based on the *AthmiR395* target genes (*AthSULTR2;1* and *AthATPS1, 3, 4*) to amplify and identify the putative homologous genes in tobacco.

**Figure 7:**
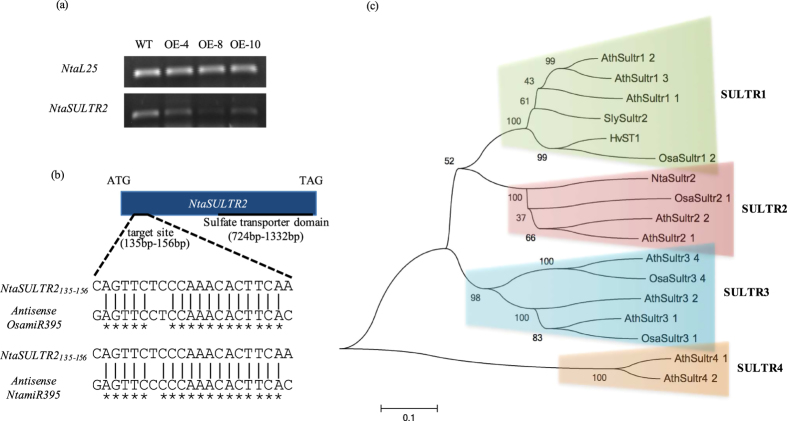
Identification of a sulfate transporter gene, *NtaSULTR2*, the target of *miR395* in tobacco. (**a**) RT-PCR analysis of *NtaSULTR2* expression in tobacco. Total RNA samples were prepared from two weeks old wild type and transgenic tobacco and used for RT-PCR analysis. *NtaL25* was used as a reference gene. Experiment was repeated three times. (**b**) General structure of tobacco gene *NtSULTR2*. *NtaSULTR2* with a length of 1335 bp contains a sulfate transporter domain between 724 bp to 1332 bp, and a *miR395* target site between 135 bp to 156 bp. The target site was compared with the complementary sequence of mature *OsamIR395h* and *NtamiR395*. Asterisks indicate the identical sequences. (**c**) phylogenetic analysis of NtaSULTR2 protein. Protein sequences of NtaSULTR2 and 16 sulfate transporters of rice and *Arabidopsis* were used to establish phylogenetic tree with MEGA6. In this phylogenetic tree, NtaSULTR2 protein is classified into the second group of sulfate transporter subfamily together with AthSULTR2;1, AthSULTR2;2 and OsaSULTR2;1.

Using these approaches, we identified a novel gene named *NtaSUTLR2* to be a putative target of *OsamiR395h* (Fig. 7). Semi-quantitative RT-PCR analysis revealed that *NtaSULTR2* was significantly down-regulated in transgenic tobacco (Fig. 7a). We cloned the full-length cDNA sequence of *NtaSULTR2* using RACE (Rapid Amplification of cDNA Ends) method, and identified the target site of *miR395* that is located between 135 bp and 156 bp of its coding region. There are four mismatches and three mismatches between *NtaSULTR2* target sequence and mature *OsamiR395* and *NtamiR395*, separately (Fig. 7b), indicating that *NtaSUTLR2* should be efficiently regulated by *miR395* because of their near perfect complementary sequence.

We further characterized *NtaSULTR2* by generating a phylogenetic tree using protein sequence of *NtaSULTR2* and other sixteen well-studied sulfate transporters from rice and *Arabidopsis* using MEGA6. In this phylogenetic tree, NtaSULTR2 protein is classified into the second group of sulfate transporter subfamily together with AthSULTR2;1, AthSULTR2;2 and OsaSULTR2;1 proteins (Fig. 7c). The three sulfate transporters from *Arabidopsis* and rice are low-affinity sulfate transporters and involved in the inter-organ delivery of sulfate in vascular to transport sulfate from root to leaf, and distribution of sulfate between leaves^4^,^7^,^8^.

Taken together, our results indicate that overexpression of *OsamiR395h* in tobacco represses sulfate transporter *NtaSULTR2*, which may play an important role in sulfate transportation and distribution, thus interrupting sulfate homeostasis and distribution in transgenics.

### Sulfate regulates tobacco *NtamiR395* and *NtaSULTR2*

To confirm that *NtaSULTR2* is the target of *miR395* in tobacco, we investigated the expression level of both *NtaSULTR2* and mature *NtamiR395* under different sulfate concentrations.

**Figure 8:**
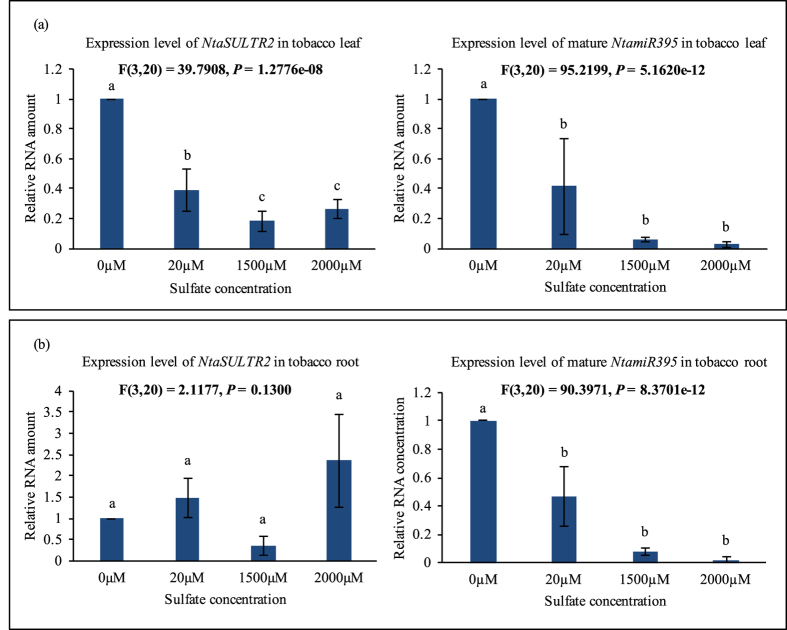
*NtamiR395* and *NtaSULTR2* exhibit opposite expression patterns in tobacco roots. Real-time PCR analysis of expressions of *NtaSULTR2* and mature *NtamiR395* under different sulfate concentrations. Total RNA samples were prepared from (**a**) leaf tissue and (**b**) root tissue of four weeks old tobacco grown in MS medium with 0, 20, 1500 or 2000 μM (NH^4+^)_2_SO_4_. *NtaL25* was used as a reference gene. Data are presented as means of three technical replicates and two biological replicates, error bars represent SD (n = 6). The statistically significant difference between groups was determined by one-way ANOVA (F(df_between_, df_within_) = F ration, *p* = p-value, where df = degrees of freedom). Means not sharing the same letter are statistically significantly different (P < 0.05).

In leaf tissues, the transcription of the mature *NtamiR395* was gradually up-regulated, contrary to the gradually reduced sulfate concentration. However, *NtaSULTR2* did not exhibit an opposite, but a similar expression pattern to *NtamiR395* with its lowest transcript level being under 1500 μM, but not 2000 μM (NH^4+^)_2_SO_4_ (Fig. 8a).

In root tissues, the situation was different. The transcript level of the mature *NtamiR395* increased in response to sulfate depletion, similar to that observed in leaves, whereas *NtaSULTR2* exhibited a roughly opposite, but more complex expression pattern (Fig. 8b). Compared to sulfate depletion conditions with 0 μM (NH^4+^)_2_SO_4_ supply, *NtaSULTR2 wa*s up-regulated under both 20 μM and 2000 μM (NH^4+^)_2_SO_4_, but down-regulated under 1500 μM (NH^4+^)_2_SO_4_. The results indicate that *NtaSULTR2* might be regulated by *NtamiR395* in roots but not in leaf tissues. These results correspond to the previous studies in *Arabidopsis* and rice showing that the expression level of *AthSULTR2* is opposite to that of *AthmiR395* in some, but not all plant tissues most likely due to the fact that the spatial expression pattern of *AthmiR395* does not overlap with that of *AthSULTR2;1*^18^,^19^,^30^, which could probably also explain the similar observation in tobacco from this study.

### *MiR395* mediates the cleavage of *NtaSULTR2* mRNA

To further confirm that *NtaSULTR2* is the true target of *miR395*, we conducted RLM-RACE (T4 RNA Ligase Mediated Rapid Amplification of cDNA Ends) to verify that *NtaSULTR2* transcripts are cleaved by *miR395*. We used RNA from the miR395-overexpressing transgenic tobacco plants to facilitate the detection of cleaved *NtaSULTR2* mRNA.

**Figure 9:**
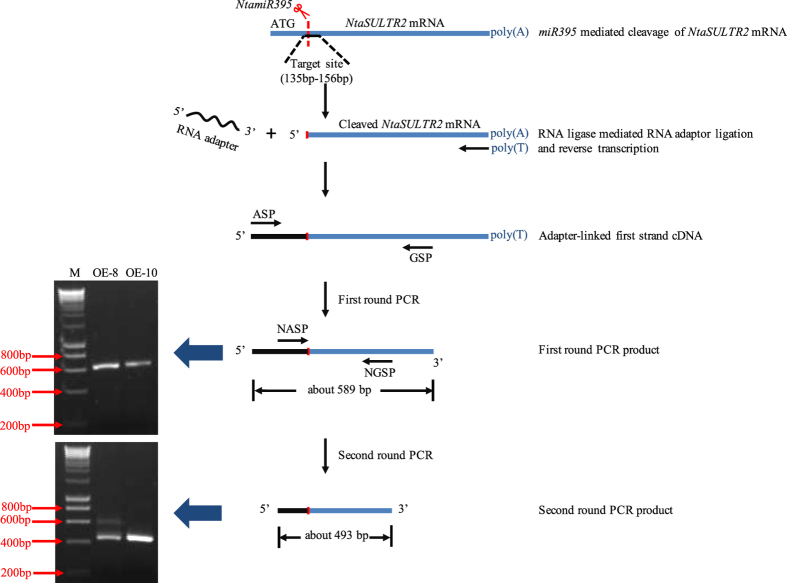
Confirmation of *miR395*-mediated cleavage of *NtSULTR2* mRNA. RLM-RACE (T4-RNA ligase mediated amplification of 5′ cDNA ends) was conducted to confirm the cleavage of *NtSULTR2* mRNA. Total RNA samples were isolated from two weeks old transgenic tobacco. 44 bp RNA adapter was ligated to the purified RNA by using T4 RNA ligase. Adapter-linked RNA was then used to synthesize first strand cDNA, followed by amplification of 5′ ends using the forward primer ASP and the reverse primer GSP. The 589 bp product from the first round PCR was then used as template for the second round PCR using the forward nest primer NASP and the reverse nest primer NGSP, producing a 493 bp second round PCR product. M: DNA molecular weight marker. OE: overexpression line. Red lines indicate *miR395* cutting site.

We used the forward primer ASP (Adapter Specific Primer) and the reverse primer GSP (Gene Specific Primer) to conduct the first round PCR after the adapter-linked first strand cDNA ends were generated. The RNA adapter has a length of 44 bp, and the reverse GSP is localized 545 bp downstream of the predicted *miR395* target site in the *NtaSULTR2* mRNA, so the product of the first round PCR should have a length of about 589 bp. As shown in Fig. 9, the first round PCR with transgenic tobacco cDNA indeed generated a clear band of about 600 bp.

A second round PCR was then conducted using the first round PCR product as template and a new set of primers to confirm the authenticity of the PCR product. The forward primer NASP (Nest Adapter Specific Primer) is localized on the adapter from 14 bp to 44 bp, and the reverse primer NGSP (Nest Gene Specific Primer) is localized 463 bp downstream of the predicted *miR395* target site in the *NtaSULTR2* mRNA, so the product of the second round PCR should be about 493 bp. As shown in Fig. 9, the second round PCR indeed generated a clear main band of about 500 bp as expected. Cloning and sequencing of the PCR product further confirmed the predicted *miR395* cleavage site in the *NtaSULTR2* mRNA.

## Discussion

Previous studies on *Arabidopsis miR395* have indicated its involvement in sulfate starvation response by repressing the expression of genes in sulfate transportation and assimilation pathways.

Under −S condition, the accumulation of *AthmiR395* is enhanced under low internal sulfate levels, and correlated to GSH pool, indicating that the regulation of *AthmiR395* is mediated by internal sulfate level and redox signaling in *Arabidopsis*^22^,^31^. The increased *AthmiR395* then represses the expression of *AthATPS1, AthATPS3, AthATPS4* and *AthSULTR2;1*^18^,^22^. Further study in *Arabidopsis* revealed a whole picture of how *AthmiR395* is involved in plant response to sulfate starvation. When sulfate supply is limited, the induced *AthmiR395* mediates the degradation of *ATPS* mRNA leading to the accumulation of sulfate in leaf tissues as a result of decelerated sulfate assimilation^19^. At the same time, the cleavage of *AthSULTR2;1* mRNA in shoots by *AthmiR395* results in blocked sulfate transport into new leaves from old ones^19^. Furthermore, the impaired sulfate homeostasis and reduced sulfate assimilation impact seed germination under ABA-treated condition^32^.

*MiR395* is highly conserved across species, which strongly suggests that its function in regulating plant response to nutrition, particularly sulfate supply could also be conserved during evolution. Our results in rice indicate that indeed, the transcript of mature *OsamiR395* increases under −S condition, and this change in expression might be regulated at the transcription level (Fig. 1). Computational prediction led to the identification of four putative target genes of *OsamiR395* in rice. We confirmed that *OsaSULTR2;1* and *OsaSULTR2* are regulated by *OsamiR395* in roots suggesting that they may be the *OsamiR395* target genes.

Knowledge about the functions of rice sulfate transporters is limited. Phylogenetic analysis grouped the fourteen rice sulfate transporters together with their *Arabidopsis* counterparts^11^, suggesting that they may share similar function. OsaSULTR2;1 and OsaSULTR2 may be responsible for the root-to-shoot sulfate transportation and distribution of sulfate between leaves of different ages. Our results (Fig. 2b–d) showed that the expression patterns of rice sulfate transporter genes were different from their *Arabidopsis* homologs, both *OsaSULTR2;1* and *OsaSULTR2* were reduced in leaves with the increasing sulfate concentrations. We speculate that the two sulfate transporter genes and *miR395* may be differentially expressed in different leaf tissues and thus, *OsaSULTR2;1* and *OsaSULTR2* may not be subjected to *miR395* regulation. Instead, other regulatory machineries may participate in the control of their expression in response to sulfate levels. It is likely that when rice plants are subjected to sulfate starvation, there is a need for the two sulfate transporters to be active, driving the transportation of sulfate from old leaves to younger ones to ensure plant growth and development. However, with abundant sulfate supply in the environment, there is no need for sulfate distribution to young leaves, and therefore the expression of both *OsaSULTR2;1* and *OsaSULTR2* declines.

The miRNA-mediated gene regulation mechanism emerged about 425 million years ago, which is at a very early stage of plant phylogeny prior to the divergence of monocot and dicot plants^33^. This suggests that monocot and dicot plants should have a similar miRNA-mediated gene regulation mechanism, and some highly conserved miRNA families regulating the same biological process have evolved from the same gene ancestors. Indeed, research data in the past twenty years indicate that 21 miRNA families, such as miR156 and miR399, are conserved in sequence across monocots and dicots^25^. More specifically, Zhang *et al*. found that 9 miRNA families are highly conserved^33^, 10 miRNA families are moderately conserved and 16 miRNA families including *miR395* are lowly conserved across plant species. In a later work, *miR395* family was identified in the common ancestor of all embryophytes^25^. Besides the miRNA sequences, the genes involved in miRNA and siRNA biogenesis pathways are also conserved across species. In plants, Dicer-like (DCL) is a key protein in the miRNA genesis pathway. DCL interacting with HYPONASTIC LEAVES1 (HYL1) and C2H2-zinc finger protein SERRATE (SE) in D-bodies cleaves the pri-miRNA from the base to yield a pre-miRNA with stem-loop structure, and this pre-miRNA is sliced again to yield mature miRNA^34^,^35^,^36^,^37^. Phylogenetic analysis indicated that divergence of *DCL1* gene associated with miRNA production from other *DCLs* could be traced to the time before the emergence of moss *Physcomitrella patens*^36^, indicating that *DCLs* may have the same origin and are conserved across vascular plants.

Based on previous findings, we hypothesize that miRNA biogenesis pathway in dicots could accept pri-miRNAs from monocots, and process it into mature miRNA with function. To verify our hypothesis, the full-length DNA sequence of pri-*OsamiR395h* was cloned from rice genome. The expression cassette of the CaMV35S-controlled rice pri*-OsamiR395h* was then prepared and introduced into tobacco genome. By performing small molecule northern blotting, we observed high transcript level of *miR395* in transgenic tobacco under normal condition, indicating that rice pri*-OsamiR395h* could be successfully expressed and processed into mature *miR395h* in tobacco (Fig. 4). At the same time, we also observed low level of endogenous mature *miR395* in WT tobacco, confirming that tobacco mature *miR395* is highly conserved with its rice homolog. All of the three transgenic tobacco lines exhibited impaired sulfate homeostasis and distribution (Fig. 5). Furthermore, transgenic plant had retarded growth phonotype (Fig. 6). All the facts suggest that mature *OsamiR395* functions in transgenic tobacco.

Data obtained from this research revealed that the sulfate-S contents in transgenic tobacco are higher in leaf tissue, but lower in root tissue than those in WT controls. An even more significant difference in total sulfur content was observed between WT controls and *OsamiR395h* overexpression plants (Fig. 5a,b). Besides, we also observed that sulfate distribution between leaves of different ages is impaired in transgenic tobacco plants (Fig. 5c).

To reveal the molecular mechanism underlying *miR395*-mediated plant sulfate metabolism, we studied genes impacted by excessive dose of *miR395* in transgenic tobacco, and identified a novel sulfate transporter gene *NtaSULTR2* belonging to the second group of sulfate transporter genes (Fig. 7). Based on the results of real-time PCR and RML-RACE, we verified that *NtaSULTR2* is the target gene of *miR395* (Figs 8 and 9). We believe that the repression of *NtaSULTR2* gene in transgenic tobacco plants partially impaired the sulfate homeostasis. In *Arabidopsis* shoot tissue, sulfate transporter *AthSULTR2;1* is localized in both xylem and phloem, particularly in phloem parenchyma cells surrounding sieve and companion cells, and involved in distribution of sulfur between leaves of different ages^7^,^28^. We conjecture that in tobacco shoot tissue, *NtaSULTR2*, likes its homologs in *Arabidopsis*, retrieves sulfate from mesophyll cells to xylem and phloem cells, and sulfate is transported from old leaves to young leaves. But in transgenic plants, the delivery of sulfate from old leaves to young leaves is impaired because of significantly repressed *NtaSULTR2* gene (Fig. 5c).

Although no *ATPS* gene have been identified and cloned in tobacco, we believe that there must be one or more *ATPS* gene(s) repressed in transgenic tobacco, causing interrupted sulfate assimilation. The interruption of the sulfate assimilation pathway would cause a shortage in cysteine and other sulfate metabolic products, resulting in retarded plant growth and triggering plant sulfate starvation signaling, which would promote sulfate absorption and transport into leaf tissue, and consequently a much more sulfur accumulation in leaves of transgenics than in that of WT controls (Fig. 5a,b).

## Materials and Methods

### Plant materials and growth conditions

To investigate the expression levels of *OsamiR395* and its targets in rice under different sulfate concentrations, rice seeds were surface sterilized and grown in N6 medium under 16h light/8h dark at 28 °C^38^. Sulfate salts of the N6 medium were replaced with chloride salts and supplemented with 0, 20, 1500 or 2000 μM (NH^4+^)_2_SO_4_. Sterilized rice seeds were also grown in regular N6 medium (+S) and N6 medium without SO_4_^+^ (−S) under 16 h light/8 h dark at 28 °C. Two weeks old plants were harvested for RNA isolation.

To investigate the expression patterns of *OsamiR395* and its targets in different developmental stages and tissues of rice, rice seeds were grown in soil in a greenhouse. Root and leaf samples were collected two, four and eight weeks after germination.

To investigate the expression levels of pri*-OsamiR395h*, mature *miR395* and *NtaSULTR2* in tobacco, tobacco seeds were surface sterilized and grown in MS medium under 16h light/8h dark at 22 °C^39^. To prepare MS mediums with different sulfate concentrations, sulfate salts of the MS medium were replaced with chloride salts and supplemented with 0, 20, 1500 or 2000 μM (NH^4+^)_2_SO_4_. Two weeks old and four weeks old plants were harvested for RNA isolation.

To measure total sulfate content and sulfate-S concentration in tobacco, and to determine the growth rate of tobacco, tobacco were grown in soil in a greenhouse. Four weeks old and 12 weeks old plants were collected for analysis.

### Genomic DNA and total RNA isolation, and cDNA synthesis

Plant genomic DNA was isolated following previously described method^40^.

Total RNA was isolated from 100 mg plant samples with Trizol reagent (Ambion, USA), and the genomic DNA is removed by using RNase-free DNase I (Invitrogen, USA). 2 μg total RNA was used to synthesize first strand cDNA with SuperScript III Reverse Transcriptase (Invitrogen, USA) according to manufacturer’s instructions. The first strand cDNA was used for semi quantitative RT-PCR and regular real-time PCR.

To determine the transcript level of mature *miR395*, the first-strand cDNA used for stem-loop real-time PCR was synthesized following the regular SuperScript III Reverse Transcriptase (Invitrogen, USA) mediated method, except that the oligo (dT)_20_ was replaced with *miR395* specific reverse transcription primer. Primers were all listed in Supplementary Table S1.

### Semi-quantitative RT-PCR, stem-loop and regular real-time PCR

To conduct semi-quantitative RT-PCR, first-strand cDNA samples were diluted to 0.25 times based on the concentration of the first-strand cDNA samples. The loading volume of the cDNA samples was adjusted basing on the transcript level of a reference gene.

To conduct stem-loop and regular real-time PCR, first-strand cDNA samples were diluted to 0.025 to 0.005 times based on the concentration of the first-strand cDNA samples. Both stem-loop and regular real-time PCR were performed using SYBR Green Supermix (Bio-Rad, USA) following manufacturer’s instructions, and iQ5 real-time detection system (Bio-Rad USA) was used to detect and analyze the real-time PCR result.

Stem-loop and regular real-time PCR results were determined by using ΔΔCt method. ΔCt was defined as Ct_test _− Ct_0h_, in which Ct_test_ stands for threshold cycle of one gene after treatment, and Ct_0h_ stands for threshold cycle of one gene before treatment. ΔΔCt was defined as ΔCt_reference_ − ΔCt_target_, in which ΔCt_reference_ stands for ΔCt of the endogenous gene used as a reference, and ΔCt_target_ stands for ΔCt of target gene. Finally, related expression ratio was calculated as 2^ΔΔCt^.

Primers used for semi-quantitative RT-PCR, stem-loop real-time PCR and regular real-time PCR were all listed in Supplementary Table 1.

### Small molecule Northern blotting

Small molecule northern blotting was performed following the method previously described with minor modification^41^. 10 μg total RNA denatured at 95 °C was separated in 12.5% urea-polyacrylamide gel and transferred to Hybond-N+ nylon membrane (Amersham, USA) in a Trans-Blot SD Semi-Dry Transfer Cell (Bio-Rad, USA). To prepare radiolabeled probe for detecting mature *miR395*, DNA oligonucleotide GAGTTCCCCCAAACACTTCAC was synthesized (http://www.idtdna.com/site) and labeled with γ-[32P]-ATP by using T4 polynucleotide kinase. RNA membrane was then hybridized with radiolabeled probe and detected on a phosphorimaging screen.

### Plasmid construction, bacterial strains and plant transformation

The predicted pri-*OsamiR395h* was amplified from rice genomic DNA and cloned at downstream of CaMV35S (Cauliflower Mosaic Virus 35S) promoter of binary vector pZH01, resulting in CaMV35S/*OsamiR395h*-CaMV35S/*hygromycin*^42^. This chimeric gene expression construct was then mobilized into *Agrobacterium tumefaciens* strain LBA4404 by electroporation for tobacco transformation. The *Escherichia coli* strain used in this experiment was DH5α.

The primers used for plasmid construction were all listed in Supplementary Table 1.

### Determination of total sulfur content and sulfate-sulfur concentration

For determination of total sulfur, plant samples were collected and dried for 48 h at 80 °C. Total sulfur contents in dry samples were determined as previously described^43^ sulfate-S concentration was determined following a previous method with minor modification^44^. 10 mg dry plant sample or 200 mg fresh plant sample was immersed in 1 ml 0.1 M HCl for 2 h at room temperature, followed by 20 min centrifugation at 12000 g. Clear supernatant liquid was then transferred to a 50 ml Erlenmeyer flask and made to 20 ml by water. One ml of barium chloride-gelatin reagent was added to the liquid. After 40 min (no more than 120 min), absorbance of the resulting cloudy liquid was determined at 454 nm by using a spectrometer.

### Rapid amplification of cDNA ends

To obtain 5′ cDNA end and 3′ cDNA end of *NtaSULTR2,* total RNA was extracted from 100 mg two weeks old WT tobacco with Trizol reagent (Ambion, USA) and treated with RNase-free DNase I (Invitrogen, USA) to remove genomic DNA. 1 μg total RNA was then used to amplify 5′ end and 3′ end cDNA of *NtaSULTR2* with SMARTer RACE 5′/3′ commercial kit (Clontech, USA) following the manufacture’s instruction. Then, the 5′ end and 3′ end cDNA fragments were sequenced. Sequence information was used to design primers for cloning of full-length *NtaSULTR2* cDNA.

The primers used for RACE and for cloning of full length *NtaSULTR2* cDNA were all listed in Supplementary Table S1.

### T4-RNA ligase mediated amplification of 5′ cDNA ends

To verify *miR395* cleavage site within *NtaSULTR2*, T4-RNA ligase mediated amplification of 5′ cDNA ends was conducted following a previously described method^45^. Briefly, total RNA was isolated from 100 mg plant sample using Trizol reagent (Ambion, USA), followed by purification of RNA with RNeasy mini kit (Qiagen, Germany). RNA adapter was ligated to the purified RNA by using T4 RNA ligase (New England Biolabs, USA). Based on the fact that miRNA-mediated mRNA cleavage will generate 5′-monophosphate ends on the 3′ end cleavage product of the target mRNAs, it is possible to ligate RNA oligonucleotide adapter to the 5′ terminus of the 3′ end cleavage product by using T4 RNA ligase, whereas such RNA oligonucleotide adapter would not be ligated to mRNAs with conventional 5′ cap^45^. Adapter-linked RNA was then used to synthesize first strand cDNA with SuperScript II Reverse Transcriptase (Invitrogen, USA), followed by amplification of 5′ ends using the forward primer ASP and the reverse primer GSP. The product from the first round PCR was then used as template for the second round PCR with the forward nest primer NASP and the reverse nest primer NGSP. PCR product was cloned for sequencing.

The primer sequences used for RML-RACE were all listed in Supplementary Table 1.

### Phylogenetic analysis of sulfate transporters

Phylogenetic tree of *NtaSULTR2* and other sulfate transporter genes in rice and *Arabidopsis* inferred using the Neighbor-Joining method^46^. The optimal tree with the sum of branch length = 3.89795523 is shown. The tree is drawn to scale, with branch lengths in the same units as those of the evolutionary distances used to infer the phylogenetic tree. The evolutionary distances were computed using the Poisson correction method^47^ and are in the units of the number of amino acid substitutions per site. The analysis involved 17 amino acid sequences. All positions containing gaps and missing data were eliminated. There was a total of 347 positions in the final dataset. Evolutionary analyses were conducted in MEGA6 48. WT: wild type plant. OE: overexpression line.

### Statistical analysis

Student’s t test was used to test the difference between the means from two groups. P < 0.05 was considered to be statistically significant and marked as *P < 0.01 was considered to be statistically highly significant and marked as**.

One-way ANOVA (F(df_between_, df_within_) = F ration, *p* = p-value, where df = degrees of freedom) with post hoc comparisons using the Tukey HSD test was used to determine the statistically significant difference between the means from three or more groups. Means not sharing the same letter are statistically significantly different (P < 0.05).

## Additional Information

**Accession codes:** *AthSULTR2;1*: NM_121056.2, *AthATPS1*: NM_113189.4, *AthATPS3*: U06275.1, *AthATPS4*: AT5G43780, *OsaSULTR2;1*: NM_001055792, *OsaSULTR2*: NM_001055793, *OsaSULTR3;4*: Os06g0143700*, OsaATPS*: NM_001057769, *OsaSiz1*: Os05g0125000, *NtaL25*: L18908, *NtaSULTR2*: KT373983.

**How to cite this article**: Yuan, N. *et al*. Heterologous expression of a rice *miR395* gene in *Nicotiana tabacum* impairs sulfate homeostasis. *Sci. Rep.* **6**, 28791; doi: 10.1038/srep28791 (2016).

## Supplementary Material

Supplementary Information
