# Supplementary material for: Heterologous expression of a rice miR395 gene in Nicotiana tabacum impairs sulfate homeostasis
Source: Sci Rep. 2016 Jun 28;6:28791. doi: 10.1038/srep28791 (PMC4923854; doi:10.1038/srep28791)
Supplement: Supplementary Information [file srep28791-s1.doc]

Heterologous expression of a rice *miR395* gene in Nicotiana tabacum impairs sulfate homeostasis

Ning Yuan (ningy@clemson.edu), Shuangrong Yuan (syuan@clemson.edu), Zhigang Li (zhiganl@clemson.edu), Dayong Li (dyli@genetics.ac.cn), Qian Hu (qhu@clemson.edu), Hong Luo* ([hluo@clemson.edu](mailto:hluo@clemson.edu))

Supplementary Table 1. Primers and probes used

| Primers for real-time and semi RT-PCR analysis |  |  |  |
| --- | --- | --- | --- |
| Gene | Primer name | Primer sequence | Note |
|  | 395_stemloop_RT | GTCGTATCCAGTGCAGGGTCCGAGGTATTCGCACTGGATACGACGAGTTC | Reverse primer for synthesis of the first strand cDNA used in stem loop real-time PCR |
| Mature miRNA395 | Osa395_stemloop_F | TCGCTGTGAAGTGTTTGGGG | Forward primer for stem loop real-time PCR detecting rice mature miRNA395 |
|  | Nta395_stemloop_F | TCGCTCTGAAGTGTTTGGGG | Forward primer for stem loop real-time PCR detecting tobacco mature miRNA395 |
|  | Universal_stemloop_R | GCAGTGGAAGGGGCATGCA | Reverse primer for stem loop real-time PCR |
| Pri-OsmiRNA395h | priOsa395h_qpcr_F132 | ACAGATCTCTCGGTTGGTGG |  |
|  | priOsa395h_qpcr_R | CTTGTTGGCACCGAGAGTTC |  |
| Rice *SIZ1* | OsaSIZ1-qPCR-F | GTGATTTGGAAGTGGTTGCG |  |
|  | OsaSIZ1-qPCR-R | ATCTCCCAGCAATCCTCATTC |  |
| Rice *SULTR2;1* | OsaSULTR21-qPCR-F-2 | TTGGAGGCACCGATACATTG |  |
|  | OsaSULTR21-qPCR-R-2 | TCTGCAAAAGCTGTCCCTATG |  |
| Rice *SULTR2* | OsaSULTR2-qPCR-F4 | TCTTCACCGTCACCTTCCTC |  |
|  | OsaSULTR2-qPCR-R4 | CTGCCATGAACCCAACGATC |  |
| Rice *ATPS* | OsAPS-qPCR-F | AATCTTCCCCTCTCCAATGC |  |
|  | OsAPS-qPCR-R | ACAGGTCCCTCTTTTCAGTTG |  |
| Rice *SULTR3;4* | OsSULTR3-qPCR-F | GGCTGTTAATTTGTTCGCGTG |  |
|  | OsSULTR3-qPCR-R | GAGATCAGCACCCGGAGTTA |  |
| Tobacco *L25* | NtaL25_qPCR_F | CCTCGTATTAGTGCACCTGGA |  |
|  | NtaL25_qPCR_R | CAGCCTTGATGTCCACAATGA |  |
| Tobacco *SULTR2* | NtaSULTR2_QPCR_F | CAACTCTTCCAACTTTGGTTG |  |
|  | NtaSULTR2_QPCR_R | TCAGGTTGGAAAACAGGCCTG |  |
| Primers for cloning of pri-OsmiRNA395 |  |  |  |
| pri-OsmiR395 | miR395h_6XbaF | TCTAGAGCAGGTCATCCTCTTCAAGT | XbaI label forward primer |
|  | miR395h_1212Sal530R | GTCGACCATCAAACGTGGCATATGA | SalI label forward primer |
| Primers for cloning of full length NtaSULTR2 cDNA |  |  |  |
|  | Ntasultr2_5’GSP | GGCAGCTTGAAAAGTACCCGCGAAGAA | For rapid amplification of 5' cDNA end of NtaSULTR2 |
|  | Ntasultr2_5’NSP | CAGGCCTGGTGGTTCCGGCACATTTAG |  |
| NtaSULTR2 | Ntasultr2_3’GSP | TCAGAGCATTGGCTACGCGACTCTTG | For rapid amplification of 3' cDNA end of NtaSULTR2 |
|  | Ntasultr2_cDNA_F | GATGGGGGAAGATGTGCTTTTGAAC | for cloning of full length NtaSULTR2 cDNA |
|  | Ntasultr2_cDNA_R | GAGAGAATTAGTTTGCATTAAAACCTTC |  |
| Primers for RML-RACE |  |  |  |
|  | RML_RACE_RNAadaptor | CGACUGGAGCACGAGGACACUGACAUGGACUGAAGGAGUAGAAA | RNA adaptor for RML-RACE |
|  | RML_RACE_ASPF | CGACTGGAGCACGAGGACACTGA | Forward primer for RML-RACE |
| NtaSULTR2 | RML_RACE_NASPF | GGACACTGACATGGACTGAAGGAGTA | Forward nest primer for RML-RACE |
|  | NtaSULTR2_GSPR | AGCACGAGTTTTGTATATGCAGCT | Reverse primer for RML-RACE |
|  | NtaSULTR2_NGSPR | CAGCAACTGGTCCAATTGCTAT | Reverse nest primer for RML-RACE |
| Probe for small RNA northern blot |  |  |  |
| Mature miR395 | Osami395_probe21 | GAGTTCCCCCAAACACTTCAC | probe used for small RNA northern blotting |
